# Inflammatory Potential of the Diet and Incidence of Crohn’s Disease and Ulcerative Colitis in the EPIC-Spain Cohort

**DOI:** 10.3390/nu13072201

**Published:** 2021-06-26

**Authors:** Marcela Guevara, Elena Salamanca-Fernández, Estrella Miqueleiz, Diana Gavrila, Pilar Amiano, Catalina Bonet, Miguel Rodríguez-Barranco, José María Huerta, Luis Bujanda, María José Sánchez, María Dolores Chirlaque, Antonio Agudo, Eva Ardanaz, Jesús Castilla

**Affiliations:** 1 Instituto de Salud Pública de Navarra, 31003 Pamplona, Spain; estrella.miqueleiz.autor@navarra.es (E.M.); me.ardanaz.aicua@navarra.es (E.A.); jcastilc@navarra.es (J.C.); 2 Consortium for Biomedical Research in Epidemiology and Public Health (CIBERESP), 28029 Madrid, Spain; diana.gavrila@carm.es (D.G.); epicss-san@euskadi.eus (P.A.); miguel.rodriguez.barranco.easp@juntadeandalucia.es (M.R.-B.); jmhuerta.carm@gmail.com (J.M.H.); mariajose.sanchez.easp@juntadeandalucia.es (M.J.S.); mdolores.chirlaque@carm.es (M.D.C.); 3 Navarra Institute for Health Research (IdiSNA), 31008 Pamplona, Spain; 4 Instituto de Investigación Biosanitaria de Granada (ibs.GRANADA), 18012 Granada, Spain; esalamanca@ugr.es; 5 Department of Epidemiology, Murcia Regional Health Council, IMIB-Arrixaca, 30008 Murcia, Spain; 6 Sub-Directorate for Public Health and Addictions of Gipuzkoa, Ministry of Health of the Basque Government, 20013 San Sebastian, Spain; 7 Epidemiology and Public Health Area, Biodonostia Health Research Institute, 20014 San Sebastian, Spain; 8 Unit of Nutrition and Cancer, Catalan Institute of Oncology—ICO, and Nutrition and Cancer Group, Epidemiology, Public Health, Cancer Prevention and Palliative Care Program, Bellvitge Biomedical Research Institute-IDIBELL, 08908 L’Hospitalet de Llobregat, Spain; cbonet@iconcologia.net (C.B.); a.agudo@iconcologia.net (A.A.); 9 Andalusian School of Public Health (EASP), 18011 Granada, Spain; 10 Department of Liver and Gastrointestinal Diseases, Biodonostia Health Research Institute, 20014 San Sebastian, Spain; LUIS.BUJANDAFERNANDEZDEPIEROLA@osakidetza.eus; 11 Department of Gastroenterology, University of the Basque Country (UPV/EHU), 20018 San Sebastian, Spain; 12 Consortium for Biomedical Research in Hepatic and Digestive Diseases, CIBEREHD, 28029 Madrid, Spain; 13 Department of Preventive Medicine and Public Health, University of Granada, 18071 Granada, Spain; 14 Departamento de Ciencias Sociosanitarias, Facultad de Medicina, Universidad de Murcia, 30100 Murcia, Spain

**Keywords:** Crohn’s disease, ulcerative colitis, inflammatory bowel disease, inflammatory potential of the diet, inflammation, prospective cohort study

## Abstract

Diet may influence the development of inflammatory bowel disease through the modulation of inflammation. We investigated whether the inflammatory potential of the diet is associated with the risk of Crohn’s disease (CD) and ulcerative colitis (UC) in the Spanish cohort of the European Prospective Investigation into Cancer and Nutrition (EPIC-Spain). The study included 32,633 participants aged 29–69 years. The inflammatory potential of the diet was measured by using an inflammatory score of the diet (ISD) based on a baseline dietary history questionnaire. Cox regression was used to estimate hazard ratios (HRs) and 95% confidence intervals (CIs). During 21 years (674,547 person-years) of follow-up, 32 and 57 participants developed CD and UC, respectively. In multivariable analysis, a one-standard deviation (SD) increment in the ISD (two-unit increase) was associated with a higher risk of CD (HR of 1.71; 95% CI: 1.05–2.80; *p* = 0.031). By contrast, ISD was not associated with UC (HR for one-SD increment of 0.89; 95% CI: 0.66–1.19; *p* = 0.436). Our results suggest that consuming a more pro-inflammatory diet may contribute to the risk of CD, supporting that a healthy diet might be beneficial in its prevention. Further, larger studies are needed to verify these findings.

## 1. Introduction

Inflammatory bowel disease (IBD) denotes a group of idiopathic immune-mediated disorders, characterized by chronic relapsing-remitting inflammation of the digestive tract. The two main types of IBD are ulcerative colitis (UC), which is a non-transmural disease limited to the colonic mucosa, and Crohn’s disease (CD), which is transmural and can cause inflammation throughout the entire gastrointestinal tract [1]. In Europe, the incidence of UC ranges between 0.9 and 24.3 per 100,000 person-years, and the estimates for CD range from 0.5 to 10.6 cases per 100,000 person-years [2]. These lifelong diseases often impose a meaningful impact on the quality of life for the affected individuals, also leading to substantive costs to the health care systems and society [3].

IBD has been regarded as a disease of industrialized societies. Its incidence increased steadily in developed countries in the last decades of the twentieth century, and it is now rising rapidly in newly industrialized countries [4]. The worldwide spread of the disease is believed to be associated with the Westernization of diets and other environmental factors, which raise the risk of IBD in persons with genetic susceptibility [4,5].

The possible effect of diet on the development of IBD is of great interest as it can be one of the risk factors most likely to be modifiable, and therefore a possible target for prevention. Available evidence suggests that diet influences gut inflammation through different mechanisms, which include alterations in the composition of gut microbiota and its interactions with the local immune system [6,7]. Several epidemiological studies have reported relationships between specific foods or nutrients and the risk of IBD, including a positive association for n-6 polyunsaturated fatty acids (PUFAs) [8,9] and animal protein intake [10], and an inverse association for n-3 PUFAs [9,11], fibre, fruit, and vegetable intake [12,13]. Some studies that have assessed the association between the overall diet (i.e., dietary patterns) and the risk of IBD have found a positive and an inverse association for unhealthier (e.g., Western) and healthier diets (e.g., Mediterranean), respectively [14,15,16]. However, very few studies have specifically evaluated the relation between the overall diet’s inflammatory potential and IBD risk. In a prospective study, a pro-inflammatory diet was related to an increased risk of CD but not UC [17], while association with UC has been observed in two case-control studies [18,19].

A widely used approach to measure the inflammatory capacity of the diet has been the Dietary Inflammatory Index (DII) [20], which was developed on the basis of an extensive literature review and has been validated against different markers of inflammation, such as homocysteine, interleukin-6 and C-reactive protein [21,22]. For the present study, we used the Inflammatory Score of the Diet (ISD), an adaptation of the DII previously described within the European Prospective Investigation into Cancer and Nutrition (EPIC) cohort [23,24,25,26]. The DII and the ISD have been found to be associated with several health-related outcomes, including cancer [24,25,26,27,28], cardiovascular diseases [29], and mortality [23,30].

The objective of this study was to evaluate the association between the inflammatory potential of the diet, as measured by the ISD, and the incidence of UC and CD among participants of the Spanish EPIC cohort.

## 2. Materials and Methods

### 2.1. Study Design and Population

EPIC is a multi-centre cohort study, conducted in 23 centres across 10 European countries, aiming to investigate the aetiological role of biological, lifestyle, and environmental factors in cancer and other chronic diseases. The full methodological details have been described elsewhere [31]. The population of the present study consisted of the participants from four Spanish centres of the EPIC cohort (Gipuzkoa, Granada, Murcia, and Navarra), which included 32,895 healthy volunteers of different social and educational levels, ~60% recruited among blood donors, 62% women, aged 29–69 years. At recruitment, between 1992 and 1996, data concerning diet, physical activity, anthropometric measurements, and medical history were obtained [32]. Follow-up for incident IBD and vital status started at the date of recruitment and ended on 31 December 2014 (in Granada and Murcia) or 31 December 2016 (in Navarra and Gipuzkoa).

Participants with extreme values of the ratio energy intake to estimated energy requirement, defined as >3 standard deviations (SD) from the average log-transformed ratio, were considered to have implausible dietary data, hence they were excluded from the study (*n* = 218 participants). In addition, we excluded 4 and 10 participants with prevalent CD and UC at recruitment, respectively. Thus, the final number of individuals included was 32,663.

The International Agency for Research on Cancer (IARC) Ethics Committee approved the EPIC study protocol. All participants gave written informed consent.

### 2.2. Exposure Measurements: Diet and Inflammatory Score of the Diet -ISD-

Participants were interviewed at recruitment to collect information on habitual diet during the previous year by using a validated dietary history questionnaire [33]. The questionnaire included 662 common foods and recipes. Taking into account seasonal variability, the frequency and amount of foods eaten at least twice a month was collected. Based on the standardised EPIC Nutrient Database [34], total energy (kilocalories/day) and nutrient intakes (grams/day) were calculated.

**Figure 1:**
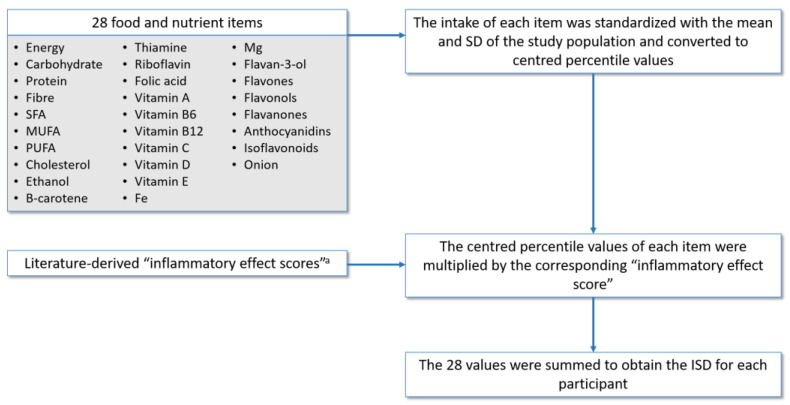
Construction of the inflammatory score of the diet (ISD). SFA: saturated fatty acids, MUFA: monounsaturated fatty acids, PUFA: polyunsaturated fatty acids. ^a^ The inflammatory effect scores used are those reported by Shivappa et al. [20].

The ISD was calculated to measure the inflammatory capacity of the diet. This is a relative index that allows to rate an individual’s diet on a scale from maximally anti- to pro-inflammatory. The procedure followed to calculate the ISD in the EPIC cohort [24,25], shown in Figure 1, is described in more detail in the page 1 of Supplementary Materials. Briefly, it was similar to the procedure for calculating the DII [20] but with some variations as explained below.

The DII is an index that includes 45 food parameters which were identified as having anti- or pro-inflammatory properties based on an extensive literature review. Each food parameter was assigned an inflammatory effect score according to its association with biomarkers [20]. Of the 45 possible items in the DII, after excluding total fat to avoid redundancy with other fats components, 28 foods parameters were available in the EPIC cohort. The intake of each item was standardized with the mean and SD of the study population, unlike the DII that uses the mean and SD of a global composite data set. Supplementary Table S1 shows the inflammatory effect scores and mean with SD of intake in the EPIC-Spain population, for each food parameter used to calculate the ISD.

The inflammatory effect scores (weights) for each food parameter were used as reported for the DII [20], with a variation in alcohol weight. Alcohol is considered to have an anti-inflammatory effect and therefore it is weighted negatively. However, an inverse association with inflammatory markers has been found only in moderate consumers [35,36]. Accordingly, we assigned a weight of zero for alcohol intakes higher than 40 g/day. 

### 2.3. Assessment of Other Covariates

Baseline data on the participants’ socio-demographic and lifestyle factors were collected by standardized questionnaires [31,32]. For the present study, we used data concerning smoking status, highest attained educational level, and combined recreational and household physical activity [37]. Weight and height, from which body mass index (BMI) was calculated, were measured at recruitment following a standardized procedure.

### 2.4. Outcome Measurements: Crohn’s Disease and Ulcerative Colitis

The cohort was followed-up to identify those participants who developed IBD by linkage with hospital discharge and primary care databases and mortality registries, using the codes for CD and UC of the International Classification of Diseases—9th revision: 555 and 556, and 10th revision: K50 and K51—and the code D94 (chronic enteritis or ulcerative colitis) of the International Classification of Primary Care-2. For each potential case, the clinical records were thoroughly reviewed by local physicians to verify the diagnoses. Participants for whom the diagnosis of IBD was not confirmed were included in the analyses as non-cases.

### 2.5. Statistical Analysis

Standard descriptive statistics were used to summarize the baseline characteristics of the cohort, and differences across ISD tertiles were tested by Chi-squared or Kruskal–Wallis rank sum tests, as appropriate. Incidence rates of CD and UC were calculated and directly age- and sex-standardized using the 2013 European Standard Population. Cox proportional hazards regression models were fitted to assess the association of the ISD with CD and UC. Age was used as the underlying timescale, with entry time defined as the participant’s age at recruitment, and exit time as the age at IBD diagnosis, censoring, or death (whichever came first). The ISD was assessed as a continuous variable (1-SD increase) and as a categorical variable according to tertiles, using the highest tertile (most pro-inflammatory diet) as the reference. Linear trends across tertiles were tested by using the median value for each tertile as a continuous variable.

Hazard ratios (HRs) and 95% confidence intervals (CIs) were obtained, adjusted for age, as time scale, and sex, and stratified by centre (model 1). A second model was additionally adjusted for energy intake as a continuous variable (model 2). Smoking status and physical activity did not change the HR by more than 10%, so they were not retained in the final model to avoid overfitting given the small number of cases. However, these covariates were retained in sensitivity analyses. The possibility of a nonlinear association of ISD with CD and UC risk was assessed by adding a quadratic term for ISD to the model. There was no evidence of a nonlinear relationship, and so a quadratic term was not retained in any of the fitted models. Effect modifications by age group (<50 and ≥50 years) and sex were evaluated by modeling interaction terms between these variables and ISD. To account for pre-diagnosis symptoms potentially influencing diet prior to diagnosis, we performed a lag analysis excluding the first two years of follow-up. For all models, the proportional hazards assumption was satisfied, evaluated by plotting the Schoenfeld residuals against the time variable in each model. 

Statistical tests were two sided, and *p*-values below 0.05 were considered significant. The analyses were performed using STATA 15.1 (Stata Corporation, College Station, TX, USA).

## 3. Results

A total of 32,663 participants, 20,168 (61.7%) women and 12,495 (38.3%) men, were included in the study. The median age at recruitment was 48.9 (interquartile range (IQR) 42.9–56.0) years. The inflammatory capacity of the diet in the cohort, as assessed by the ISD, had a mean (±SD) value of 0.24 (±2.02) and a median of 0.35, ranging from −6.08 to 4.98.

The main baseline characteristics of the cohort by tertiles of the ISD are shown in Table 1. Participants in the highest ISD tertile (more pro-inflammatory diet) had a lower energy intake and a higher percentage of women, physically inactive persons and never-smokers compared with those in the lowest tertile (less inflammatory diet). During an average follow-up period of 20.7 years, totaling 674,547 person-years at risk, 32 participants (56% women) developed incident CD at a median age of 60.7 (IQR 51.9–70.1) years. The median time between recruitment and diagnosis was 11.2 years (range 0.1–19.7 years). A further 57 participants (51% women) developed incident UC at a median age of 59.0 (IQR 54.7–65.1) years, with a median time between recruitment and diagnosis of 12.5 years (range 0.1–21.3 years). The crude incidence rate of CD was 4.7 (95% CI: 3.1–6.4) cases per 100,000 person-years and the age- and sex-standardized incidence rate was 2.3 (95% CI: 1.2–3.3) per 100,000 person-years. The corresponding rates of UC were 8.5 (95% CI: 6.3–10.6) and 7.1 (95% CI: 1.9–12.4) cases per 100,000 person-years.

### 3.1. ISD and Risk of Crohn Disease

Among the 32 participants who developed CD, 12 had an ISD value below the cohort average. In multivariable Cox regression analysis, after adjustment by age, sex and energy intake and stratified by centre, one-SD increment in the ISD (equivalent to two-unit increase) was associated with a higher risk of CD with HR of 1.71 (95% CI: 1.05–2.80, *p* = 0.031). The HR for the lowest tertile was 0.32 (95% CI: 0.10–1.05) as compared with the highest tertile (*P*-trend = 0.067) (Table 2). Exclusion of the first two years of follow-up and the additional adjustment for smoking status and physical activity yielded similar results (Table 3). We did not observe a statistically significant interaction between ISD and sex (*P* for interaction = 0.234), nor between ISD and age group (*P* for interaction = 0.676).

### 3.2. ISD and Risk of Ulcerative Colitis

Cox regression analyses showed no association between ISD and the risk of UC. The HR for a one-SD increment in ISD was 0.89 (95% CI: 0.63–1.26, *p* = 0.515) (Table 2). The sensitivity analyses showed similar results (Table 3). No interactions were found with sex or age group (both P for interaction >0.32). 

## 4. Discussion

In this prospective cohort study in Spanish adults, we found that higher ISD values (i.e., most pro-inflammatory diets) were associated with an increased risk of CD, independently of age, sex, energy intake, and centre when ISD was examined as a continuous measure. However, no significant association was observed when ISD was analysed as a categorical variable, possibly due to the small number of cases in each category. On the other hand, no association was found between ISD and risk of developing UC. The finding of a possible association between inflammatory diet and UC is particularly relevant given the increasing incidence of this disease worldwide [4], and since the modifiable nature of diet offers the possibility of prevention.

Supplementary Table S2 shows a summary of previous studies on the association between the inflammatory potential of the diet and risk of IBD. To our knowledge, only one previous prospective study, by Lo et al., has specifically investigated the association between the inflammatory potential of the diet and the risk of CD and UC [17]. They performed a pooled analysis of three large US cohorts, using an empirical dietary inflammatory pattern score, and found a positive association between a pro-inflammatory diet and CD risk (51% higher risk for the highest quartile vs. lowest), but no association with UC [17], results that are in line with ours. In contrast, two Iranian case-control studies, using the DII or a modified version of it, found a positive association between a more inflammatory diet and the risk of UC [18,19]. However, potential limitations of these studies include the possibility of recall and selection biases, which are inherent in most case-control studies. 

There have also been some studies examining the effect of the inflammatory potential of the diet, as measured by a score, on gut inflammation and disease activity in patients already diagnosed with IBD, but both positive and null relationships have been reported [38,39].

Dietary patterns in relation to biomarkers of inflammation have been investigated in several epidemiological studies [40,41,42,43,44]. Dietary patterns grouped under the term “Western” diet, including high-fat, high-sugar, high-protein or frequent consumption of red and processed meat and low intake of fruit and vegetables, tend to be positively associated with biomarkers of inflammation [40,41,42,45]. On the other hand, “healthy” patterns, including the Mediterranean-style diet, usually characterized by higher intakes of fruit, vegetables, legumes, fish, poultry, and whole grains, have been shown to be inversely associated with inflammatory biomarkers [40,43,44,45].

Evidence also suggests that Western-type diets are positively whereas healthy patterns are inversely associated with the risk of IBD. For instance, an analysis performed in other EPIC sub-cohorts showed that a dietary pattern characterized by high consumption of sugar and soft drinks, and low intake of vegetables, was linked to an increased risk of UC [14]. Moreover, a recent meta-analysis concluded that a Western dietary pattern might increase the risk of CD and UC [46]. In a study conducted in two prospective cohorts, higher adherence to a Mediterranean diet was associated with a decreased risk of later-onset CD [16]. Similarly, a previous case-control study performed in children showed that a Mediterranean-style diet (rich in vegetables, fruit, grains, olive oil, fish, and nuts) was linked to a decreased risk of CD, whereas a Western dietary pattern (higher intake of fatty foods, meats, and desserts) was associated with an increased risk [15]. Taken together, these studies indirectly support the hypothesis of a relationship between the inflammatory effects of the diet and the risk of IBD. However, unlike our study, they were not focused on inflammation and therefore the studied dietary patterns were not defined based on the inflammatory properties of the diet. By using an index constructed on the basis of known associations of foods or nutrients with inflammation markers [20], we provide more direct insight concerning the relationship between the inflammatory potential of the diet and IBD risk.

A number of plausible explanations have been proposed to elucidate how the inflammatory potential of diet contributes to the inflammatory process underline the IBD aetiology, although the exact pathophysiological mechanisms are yet to be completely understood. It is well known that diet is a key factor in determining the structure and activity of the human gut microbiome [7,47]. A mounting body of evidence indicates that gut microbiome and their metabolites play a pivotal role in the function and integrity of the gastrointestinal tract, as well as in the maintenance of local and systemic immune homeostasis [6,7]. Various microbial metabolites have an important effect on the regulation of the immune system through host receptors and other target molecules, and changes in these metabolites can alter immune and inflammatory responses [7,48]. Short- and long-term dietary patterns can modify the composition of the gut microbiome. For instance, Western type diets have been shown to reduce the gut microbial diversity, leading to poor production of beneficial microbial metabolites like short-chain fatty acids, which exert diverse effects on the maintenance of homeostasis [5]. Furthermore, these diets can promote the expansion of colonic mucus-degrading bacteria which results in barrier dysfunction. These alterations have been reported in IBD patients [49]. The opposite effects have been observed for plant-based diets, rich in dietary fibre [5]. According to all this evidence, probable mechanisms for the observed association between the ISD and CD include the effect of pro-inflammatory diets on gut mucosal inflammation through alterations of the gut microbiome and the epithelial barrier function.

Our study has a number of limitations. The small number of cases in the cohort, due to the relatively low incidence of IBD, limited the statistical power for some analyses. Children and very young adults were not represented in the study. Dietary data were collected at a single time point (at baseline), so we were not able to account for variations in dietary intake over time and this may have led to an underestimation of the associations. Although we used a previously validated dietary history questionnaire [33], and we excluded from the analysis participants reporting implausible diets, measurement error in self-reported dietary intake may be present. The ISD tends to have higher values at lower energy intake, as a methodological limitation related to the construction of the score. Consequently, the ISD was higher among women, who have a lower caloric intake, and who in turn are less likely to be smokers. Therefore, we included sex and energy intake among the covariates in the main model to control for potential confounding, and smoking status was included in sensitivity analyses that showed similar results. Participants with undiagnosed IBD would have been considered as non-cases, but this misclassification would have attenuated the effect estimates. Finally, as inherent to all observational designs, residual confounding from unknown or unmeasured factors, such as early-life antibiotic exposure, cannot be completely ruled out.

Strengths of the current study include the prospective longitudinal design that avoids the possibility of reverse causation bias present in cross-sectional studies, the large study population involving participants from different Spanish geographic areas, and the long follow-up period that allowed accumulating a sufficient number of cases to detect a possible association for Crohn’s disease. Additional studies are needed to confirm this association, and in this respect, our results may contribute to future meta-analyses of the emerging literature on this topic. Other study strengths are the comprehensive evaluation of dietary intake, the availability of sociodemographic, anthropometric, and lifestyle data to adjust for, and the inclusion of only verified incident cases. Moreover, the ISD has been shown to be a valid tool in predicting different health outcomes [23,24,25,26].

## 5. Conclusions

Our results suggest an association between a more pro-inflammatory diet, as measured by the ISD, and higher risk of developing Crohn’s disease. Our findings are consistent with the available literature showing a potential protective effect of consuming a diet rich in anti-inflammatory foods (such as vegetables, fruits, and legumes) and reducing the pro-inflammatory dietary intake (such as red meat, processed meat, saturated fats, and sugar) on the risk of Crohn’s disease. Studies using alternative methods for measuring the dietary inflammatory potential would provide further insight regarding this association and contribute to the verification of these findings.

## Figures and Tables

**Table 1:** Baseline characteristics of the study participants according to tertiles of the Inflammatory Score of the Diet (ISD).

	All Participants	Inflammatory Score of the Diet Tertiles ^a^			*p*-Value ^b^
		Tertile 1	Tertile 2	Tertile 3	
Total cohort, n	32,663	10,888	10,888	10,887	
Sex, *n* (%)					<0.001
Women	20,168 (61.7)	4513 (41.4)	6796 (62.4)	8859 (81.37)	
Men	12,495 (38.3)	6375 (58.5)	4092 (37.6)	2028 (18.6)	
Age at recruitment [years], *n* (%)					<0.001
<40	4171 (12.8)	968 (8.9)	1393 (12.8)	1810 (16.6)	
40–<50	13,655 (41.8)	4785 (43.9)	4627(42.5)	4243 (39.0)	
50–<60	10,665 (32.6)	3886 (35.7)	3566 (32.8)	3213 (29.5)	
≥60	4172 (12.8)	1249 (11.5)	1302 (12.0)	1621 (14.9)	
Body mass index [kg/m^2^], mean (SD)	28.3 (4.3)	28.2 (4.0)	28.2 (4.3)	28.5 (4.6)	<0.001
Educational level, *n* (%)					<0.001
No formal education	11,965 (36.6)	3460 (31.8)	3858 (35.4)	4647 (42.7)	
Primary school	12,183 (37.3)	4199 (38.6)	4139 (38.0)	3845 (35.3)	
Technical or professional training	2637 (8.1)	1057 (9.7)	906 (8.3)	674 (6.2)	
Secondary school	1921 (5.9)	687 (6.3)	647 (5.9)	587 (5.4)	
University degree	3750 (11.5)	1436 (13.2)	1255 (11.5)	1059 (9.7)	
Not specified	207 (0.63)	49 (0.4)	83 (0.7)	75 (0.7)	
Physical activity, *n* (%)					<0.001
Inactive	13,680 (41.9)	4052 (37.22)	4495 (41.3)	5133 (47.1)	
Moderately inactive	9468 (29.0)	3017 (27.7)	3228(29.6)	3223(26.6)	
Moderately active	5881 (18.0)	2164 (19.9)	2004 (18.4)	1713 (15.7)	
Active	3634 (11.1)	1655 (15.2)	1161 (10.7)	818 (7.5)	
Smoking status, *n* (%)					<0.001
Never	18,266 (55.9)	5298 (48.7)	6089 (55.9)	6879 (63.2)	
Former	5666 (17.3)	2460 (22.6)	1905 (17.5)	1301 (11.9)	
Current	8716 (26.7)	3126 (28.7)	2890 (26.5)	2700 (24.8)	
Unknown	15 (0.1)	4 (0.0)	4 (0.0)	7 (0.1)	
Energy intake [kcal/day], mean (SD)	2174 (704)	2674 (699)	2143 (561)	1705 (461)	<0.001

^a^ First, second, and third Inflammatory Score of the Diet tertile ranges were −6.08 to −0.67, −0.67 to 1.29, and 1.29 to 4.98, respectively. ^b^ *p* values calculated from Chi-squared test for categorical variables and Kruskal-Wallis rank sum test for continuous variables.

**Table 2:** Association of the Inflammatory Score of the Diet (ISD) with incidence of Crohn’s disease and ulcerative colitis.

	ISD Tertiles ^a^				1-SD Increment in ISD (Continuous)	
	Tertile 1	Tertile 2	Tertile 3			
	HR (95% CI)	HR (95% CI)	HR (95% CI)	*P*-Trend	HR (95% CI)	*P*-Trend
**Crohn’s disease**						
No. of cases/person-years	6/225,930	13/226,335	13/222,283			
Model 1 ^b^	0.44 (0.15–1.27)	1.00 (0.48–2.23)	1 (ref.)	0.142	1.42 (0.94–2.14)	0.098
Model 2 ^c^	0.32 (0.10–1.05)	0.87 (0.38–2.01)	1 (ref.)	0.067	1.71 (1.05–2.80)	0.031
**Ulcerative colitis**						
No. of cases/person-years	23/225,930	19/226,335	15/222,283			
Model 1 ^b^	1.48 (0.71–3.06)	1.27 (0.63–2.55)	1 (ref.)	0.297	0.89 (0.66–1.19)	0.436
Model 2 ^c^	1.49 (0.66–3.34)	1.27 (0.62–2.60)	1 (ref.)	0.340	0.89 (0.63–1.26)	0.515

Abbreviations: HR, hazard ratio; CI, confidence interval; SD, standard deviation; Ref, reference category. ^a^ First, second, and third Inflammatory Score of the Diet tertile ranges were −6.08 to −0.67, −0.67 to 1.29, and 1.29 to 4.98, respectively. ^b^ Model 1 is a Cox proportional hazards regression model stratified by centre and adjusted for age (as time scale) and sex. ^c^ Model 2: Model 1 + energy intake (continuous).

**Table 3:** Sensitivity analyses of the association of the Inflammatory Score of the Diet (ISD) with incidence of Crohn’s disease and ulcerative colitis.

	ISD Tertiles ^a^				1-SD Increment in ISD (Continuous)	
	Tertile 1	Tertile 2	Tertile 3			
	HR (95% CI)	HR (95% CI)	HR (95% CI)	*P*-Trend	HR (95% CI)	*P*-Trend
**Crohn’s disease**						
Model 2 + smoking + physical activity	0.35 (0.10–1.15)	0.91 (0.39–2.10)	1 (ref.)	0.095	1.64 (1.00–2.67)	0.050
Model 2 after excluding the first 2 years of follow-up ^b^	0.36 (0.10–1.13)	0.93 (0.40–2.19)	1 (ref.)	0.085	1.72 (1.05–2.83)	0.032
**Ulcerative colitis**						
Model 2 + smoking + physical activity	1.51 (0.67–3.42)	1.28 (0.62–2.62)	1 (ref.)	0.322	0.89 (0.63–1.25)	0.498
Model 2 after excluding the first 2 years of follow-up ^b^	1.41 (0.62–3.22)	1.07 (0.51–2.26)	1 (ref.)	0.398	0.92 (0.65–1.32)	0.667

Abbreviations: HR, hazard ratio; CI, confidence interval; SD, standard deviation; Ref, reference category. Model 2 is a Cox proportional hazards regression model stratified by centre and adjusted for age (as time scale), sex and energy intake (continuous). ^a^ First, second, and third Inflammatory Score of the Diet tertile ranges were −6.08 to −0.67, −0.67 to 1.29, and 1.29 to 4.98, respectively. ^b^ One case of Crohn’s disease and 4 cases of ulcerative colitis are excluded.

## Data Availability

Data are available from the authors upon reasonable request.

## Supplementary Materials

The following are available online at https://www.mdpi.com/article/10.3390/nu13072201/s1, Table S1: Food or nutrient items, inflammatory effect scores and intake in the EPIC-Spain population used to calculate the inflammatory score of the diet (ISD), Table S2: Summary of previous studies on the association between the inflammatory potential of the diet and risk of IBD.
