# Supplementary material for: Inflammatory Potential of the Diet and Incidence of Crohn’s Disease and Ulcerative Colitis in the EPIC-Spain Cohort
Source: Nutrients. 2021 Jun 26;13(7):2201. doi: 10.3390/nu13072201 (PMC8308349; doi:10.3390/nu13072201)
Supplement: Supplementary file 1 [file nutrients-13-02201-s001.zip › nutrients-1238656-supplementary.pdf]

Construction of the Inflammatory Score of the Diet

The inflammatory Score of the Diet (ISD), previously described in detail elsewhere [1,2], was used to assess the diet's inflammatory potential. The procedure to construct this score is initially based on the literature-derived Dietary Inflammatory Index (DII) [3].

The **DII** is an index that uses the dietary intake of 45 food parameters selected because of their anti- or pro-inflammatory properties, identified through a comprehensive literature review. Each food parameter was assigned an inflammatory weighting ("*inflammatory effect score*") according to its association with six well-known inflammatory biomarkers (tumor necrosis factor, IL-1b, IL-4, IL-6, IL-10 and C-reactive protein). The algorithm used to define these weights took into account the level of evidence and the number of articles found in the literature review [3]. The DII has been validated against circulating levels of inflammatory biomarkers in different populations [4,5].

The **ISD**, used in the present study, includes the 28 food parameters (of the 45 possible in the DII) which were available in the EPIC cohort after excluding total fat to avoid redundancy with other fats components [1,2]. In order to improve internal validity, as the objective of our study was to evaluate the association between the ISD and risk of IBD in our cohort, not to compare the dietary inflammatory potential between populations, the intake of each food parameter was standardized with the mean and standard deviation (SD) of the study population, unlike the DII that uses the mean and SD of a global composite data set. In the same way as for the DII, the standardized intakes were then converted to centered percentile values and they were multiplied by the respective *inflammatory effect score* (Supplemental Table S1). The *inflammatory effect scores* for each food parameter were used as reported for the DII [3], with a variation in alcohol weight. Alcohol is considered to have an anti-inflammatory effect and therefore it has a negative weight; however, the negative association with inflammatory markers has been found only in moderate consumers [6,7], accordingly, we assigned a weight of zero for alcohol intakes higher than 40 g/d. Finally, for each participant, the values for each of the 28 dietary components were summed to obtain their ISD.

Supplemental Table S1. Food parameters, inflammatory effect scores and intake in the EPIC-Spain population used to calculate the inflammatory score of the diet (ISD).

Food parameters[a]	Inflammatory effect score[b]	Daily intake	
		Mean	Standard deviation
Energy (kcal)	0.180	2137.85	688.94
Macronutrients			
Carbohydrate (g)	0.097	217.85	70.78
Protein (g)	0.021	102.34	31.64
Fibre (g)	-0.663	24.61	8.49
Fats (components)			
Saturated fat (g)	0.373	26.52	12.31
MUFA (g)	-0.009	37.51	15.63
PUFA (g)	-0.337	13.81	7.71
Cholesterol (mg)	0.110	381.39	155.10
Other dietary components			
Ethanol (g)[c]	-0.278	13.44	22.57
Vitamins			
Vitamin A (RE)[d]	-0.401	493.52	754.87
β-carotene (μg)	-0.584	2463.49	1571.43
Thiamine (mg)	-0.098	1.48	0.52
Riboflavin (mg)	-0.068	1.70	0.60
Vitamin B6 (mg)	-0.365	2.03	0.60
Folic acid (μg)	-0.190	317.81	114.34
Vitamin B12 (μg)	0.106	6.89	4.31
Vitamin C (mg)	-0.424	148.37	80.73
Vitamin D (μg)	-0.446	4.06	2.86
Vitamin E (mg)	-0.419	13.41	6.76
Minerals			
Fe (mg)	0.032	15.41	5.48
Mg (mg)	-0.484	339.32	106.50
Flavonoids			
Flavan-3-ol (mg)	-0.415	30.23	33.40
Flavones (mg)	-0.616	14.63	11.52
Flavonols (mg)	-0.467	33.82	22.94
Flavanones (mg)	-0.250	43.84	43.56
Anthocyanidins (mg)	-0.131	34.98	43.69
Isoflavonoids (mg)	-0.593	0.07	0.32
Foods			
Onion (g)	-0.301	20.76	15.84

Abbreviations: MUFA: monounsaturated fatty acids, PUFA: polyunsaturated fatty acids.

The population used to calculate the ISD included the five Spanish centres of the EPIC-Spain cohort (Asturias, Gipuzkoa, Granada, Murcia and Navarra).

[a] The following food or nutrients items were also included in the original DII [3]: caffeine, eugenol, ginger, fat (total), n-3 fatty acids, n-6 fatty acids, trans fat, garlic, niacin, pepper, rosemary, saffron, selenium, turmeric, green/black tea, thyme/oregano, Zn.

[b] Inflammatory effect score: values reported as "overall inflammatory effect score" in the Table 2 of Shivappa et al [3].

[c] The ethanol inflammatory effect score was set to zero for intakes higher than 40 g/d.

[d] Retinol Equivalents: retinol + 1/6 β-carotene + 1/12 α-carotene.

Supplemental Table S2. Summary of previous studies on the association between the inflammatory potential of the diet and risk of IBD.

Study	Year	Design and population	Tools and outcomes	Results	Conclusions
Khademi, Z., et al. [8]	2021	-Case-control study from Iran.<br>-109 cases and 218 controls.	- Inflammatory potential of the diet (IPD) score.<br>- Risk of UC	UC cases were more likely to be in the highest quartile of IPD score compared with controls (OR: 2.83; 95% CI: 1.41–5.69, P-trend <0.001).	A pro-inflammatory diet was associated with increased risk of UC.
Lo, C.-H., et al. [9]	2020	- Prospective study from U.S.A. (Nurses' Health Study I and II, and Health Professionals Follow-up Study).<br>-166,903 women and 41,931 men (328 CD cases and 428 UC cases).	-Empirical Dietary Inflammatory Pattern (EDIP) score.<br>-Risk of CD and UC.	Increased risk of CD at the highest quartile of cumulative average EDIP score vs. the lowest quartile (HR: 1.51; 95% CI: 1.10–2.07; P-trend =0.01). No association with UC (P-trend = 0.62).	A pro-inflammatory diet was associated with increased risk of CD but not UC.
Shivappa, N., et al. [10]	2016	-Case control study from Iran.<br>-62 UC cases and 124 hospital controls.	-Dietary inflammatory index (DII).<br>- Risk of UC.	UC cases were more likely to have higher DII than controls. OR per 1-unit DII increase: 1.55; 95% CI: 1.04–2.32. Tertile 3 vs. 1 OR: 2.58, 95% CI: 1.03–6.48, P-trend = 0.04.	A pro-inflammatory diet was associated with increased risk of UC.

Abbreviations: UC, ulcerative colitis; CD, Crohn´s disease.

References

1. Agudo, A.; Cayssials, V.; Bonet, C.; Tjonneland, A.; Overvad, K.; Boutron-Ruault, M.-C.; Affret, A.; Fagherazzi, G.; Katzke, V.; Schubel, R.; et al. Inflammatory potential of the diet and risk of gastric cancer in the European Prospective Investigation into Cancer and Nutrition (EPIC) study. *Am. J. Clin. Nutr.* **2018**, *107*, 607–616.

2. Jakszyn, P.; Cayssials, V.; Buckland, G.; Perez-Cornago, A.; Weiderpass, E.; Boeing, H.; Bergmann, M.M.; Vulcan, A.; Ohlsson, B.; Masala, G.; et al. Inflammatory potential of the diet and risk of colorectal cancer in the European Prospective Investigation into Cancer and Nutrition study. *Int. J. Cancer* **2020**, *147*, 1027–1039.

3. Shivappa, N.; Steck, S.E.; Hurley, T.G.; Hussey, J.R.; Hébert, J.R. Designing and developing a literature-derived, population-based dietary inflammatory index. *Public Health Nutr.* **2014**, *17*, 1689–1696.

4. Phillips, C.M.; Shivappa, N.; Hébert, J.R.; Perry, I.J. Dietary Inflammatory Index and Biomarkers of Lipoprotein Metabolism, Inflammation and Glucose Homeostasis in Adults. *Nutrients* **2018**, *10*, 1033.

5. Shivappa, N.; Hébert, J.R.; Rietzschel, E.R.; De Buyzere, M.L.; Langlois, M.; Debruyne, E.; Marcos, A.; Huybrechts, I. Associations between dietary inflammatory index and inflammatory markers in the Asklepios Study. *Br. J. Nutr.* **2015**, *113*, 665–71.

6. Imhof, A.; Woodward, M.; Doering, A.; Helbecque, N.; Loewel, H.; Amouyel, P.; Lowe, G.D.O.; Koenig, W. Overall alcohol intake, beer, wine, and systemic markers of inflammation in western Europe: results from three MONICA samples (Augsburg, Glasgow, Lille). *Eur. Heart J.* **2004**, *25*, 2092–2100.

7. Bell, S.; Mehta, G.; Moore, K.; Britton, A. Ten-year alcohol consumption typologies and trajectories of C-reactive protein, interleukin-6 and interleukin-1 receptor antagonist over the following 12 years: a prospective cohort study. *J. Intern. Med.* **2017**, *281*, 75–85.

8. Khademi, Z.; Saneei, P.; Hassanzadeh-Keshteli, A.; Daghaghzadeh, H.; Tavakkoli, H.; Adibi, P.; Esmaillzadeh, A. Association Between Inflammatory Potential of the Diet and Ulcerative Colitis: A Case-Control Study. *Front. Nutr.* **2021**, *7*.

9. Lo, C.-H.; Lochhead, P.; Khalili, H.; Song, M.; Tabung, F.K.; Burke, K.E.; Richter, J.M.; Giovannucci, E.L.; Chan, A.T.; Ananthakrishnan, A.N. Dietary Inflammatory Potential and Risk of Crohn's Disease and Ulcerative Colitis. *Gastroenterology* **2020**, *159*, 873-883.e1.

10. Shivappa, N.; Hébert, J.R.; Rashvand, S.; Rashidkhani, B.; Hekmatdoost, A. Inflammatory Potential of Diet and Risk of Ulcerative Colitis in a Case-Control Study from Iran. *Nutr. Cancer* **2016**, *68*, 404–409.
